# The Influence of Distributed Leadership on Chinese Teachers’ Job Satisfaction: The Chain Mediation of Teacher Collaboration and Teacher Self-Efficacy

**DOI:** 10.3390/ijerph22040507

**Published:** 2025-03-26

**Authors:** Xiaodong Fan, Zuwang Chu

**Affiliations:** School of Public Administration, China University of Geosciences, Wuhan 430074, China; fanxiaodong@cug.edu.cn

**Keywords:** distributed leadership, teacher job satisfaction, teacher collaboration, teacher self-efficacy

## Abstract

The leadership structure within educational institutions has a significant bearing on teacher job satisfaction (TJS). This study employs data from the OECD’s Teaching and Learning International Survey (TALIS 2018) specific to Shanghai to construct a structural equation model that investigates how distributed leadership (DL) impacts TJS in China. Findings reveal that DL has a markedly positive effect on the job satisfaction of teachers in China. Teacher self-efficacy (TSE) serves as an independent mediator in this relationship, while teacher collaboration (TC) does not mediate independently; nonetheless, both collaborate sequentially in a mediation pathway. These findings affirm the relevance of DL in the Chinese educational setting, offering empirical evidence to inform policymakers. Furthermore, it underscores that enhancing TC and TSE can lead to increased job satisfaction under DL. The results highlight the practical implications of this study for the advancement of educational governance and the optimization of school management models in China.

## 1. Introduction

Teachers are instrumental in promoting both the academic success and the comprehensive development of students [1,2]. However, teacher attrition represents a pervasive issue within multiple national education systems [3,4,5], leading to financial strain and adversely affecting student outcomes along with the overall quality of the teaching profession [6,7,8]. While factors such as income, socioeconomic conditions, occupational stress, and employment terms influence teacher attrition [9,10,11], low teacher job satisfaction (TJS), namely, discontent with the work environment or the profession itself, is a primary driver [12,13].

Given that low teacher job satisfaction is a primary driver of attrition, understanding its broader organizational impacts becomes imperative. Teacher job satisfaction (TJS) is a cornerstone of sustainable educational systems. Research consistently demonstrates that low teacher job satisfaction directly exacerbates institutional instability and compromises student outcomes [14,15]. For instance, in East Asian contexts marked by high-stakes accountability systems, dissatisfied teachers are more likely to exit the profession, deepening workforce shortages [16]. Beyond retention, satisfied teachers actively engage in collaborative practices such as peer mentoring and curriculum co-design, which enhance pedagogical innovation through strengthened professional networks [17,18].

Given the critical role of teacher job satisfaction in fostering collaboration, the leadership structure within educational institutions emerges as a key determinant of teacher satisfaction. The leadership structure within educational institutions is a prominent determinant of teacher job satisfaction [19]. With the decentralization trend in educational organizations, conventional hierarchical leadership models are progressively yielding to distributed leadership (DL) approaches [20], which are seen as vital for boosting teacher satisfaction [21]. In DL, leadership activities and decision-making powers are not limited to a sole leader or upper echelons of management but are flexibly distributed among various organizational members according to situational needs and personal competencies [22]. This paradigm diverges from traditional bureaucratic leadership through an emphasis on empowerment and shared accountability while also valuing member engagement and contributions to enhance collective efficacy [23]. Studies show that distributed leadership enhances teacher job satisfaction by bolstering self-efficacy and fostering resource sharing and collaboration among teachers [24], indicating that teacher self-efficacy (TSE) and teacher collaboration (TC) might act as mediators between distributed leadership and teacher job satisfaction [25].

In China’s educational landscape, where reforms increasingly emphasize school autonomy amid persistent centralized governance, identifying leadership models that sustain teacher job satisfaction becomes critical for reconciling systemic control with localized adaptability [26]. The uniqueness of this context lies in the coexistence of two competing paradigms: a centralized system manifested through national curriculum standards and a unified examination system and decentralized practices emerging from school-based curricula and expanded institutional autonomy [27,28]. This inherent tension between policy centralization and operational decentralization constitutes the essential context for implementing distributed leadership in Chinese schools.

While China exemplifies the tension between centralization and decentralization, cross-national studies reveal broader cultural variations in distributed leadership implementation. A recent cross-national comparative study encompassing 40 countries underscored the broad applicability of distributed leadership across cultures while also noting substantial disparities in distributed leadership implementation among different countries [29]. Therefore, it is necessary to examine whether distributed leadership can produce positive outcomes comparable to those seen in Western settings, particularly in China and other East Asian countries such as Japan and South Korea, which are characterized by hierarchical structures, significant power distance, and a collectivist orientation [30,31]. The existing literature falls short of providing a thorough investigation into the relationship between distributed leadership and teacher job satisfaction in the Chinese context. Thus, there is an imperative need to conduct in-depth research on the influence of distributed leadership on teacher job satisfaction within this specific cultural environment.

Building on this foundation, this study utilized data from the Teaching and Learning International Survey (TALIS 2018) conducted by the OECD, focusing on teachers in Shanghai, to explore how distributed leadership directly influences teacher job satisfaction in the Chinese educational setting. Moreover, this study aimed to investigate the mechanisms by which distributed leadership affects teacher job satisfaction through the mediation of teacher collaboration and teacher job satisfaction.

## 2. Theoretical Background and Research Hypotheses

### 2.1. Distributed Leadership and Conservation of Resources Theory

Distributed Leadership (DL) operates as a structured governance model that systemizes leadership distribution through institutionalized decentralization [32]. Diverging from Shared Leadership’s reliance on transient power negotiations among individuals, DL evolves from the dynamic interaction between formal institutional frameworks and informal collaborative networks [33]. In school organizations, this manifests through teaching–research groups and disciplinary committees that establish hierarchical decision-making networks—such as principals delegating authority to grade-level coordinators who subsequently empower classroom teachers—to create stable leadership frameworks [34,35]. Complementing these structures, culturally rooted practices, including mentorship systems and collaborative pedagogical planning, allow teams to dynamically recalibrate leadership roles in response to emergent tasks, exemplified by subject specialists temporarily assuming technology coordination duties during pandemic-induced remote teaching transitions [36].

This structural duality enables DL to balance structural stability with contextual responsiveness, generating temporally differentiated outcomes [37]. Short-term effects materialize through rapid role reconfigurations addressing immediate crises, while long-term impacts emerge as sustained institutional practices—such as multi-semester teaching–research initiatives—progressively transform teachers from policy implementers into pedagogical co-designers, culminating in shared mental models across educational communities [33,38].

Building on this institutional logic, the Conservation of Resources (COR) Theory substantiates DL’s collaborative architecture through its emphasis on strategic resource orchestration. COR posits that individuals deploy defensive tactics to safeguard existing resources while proactively cultivating networks to harvest new ones [39]. Within China’s basic education ecosystem, distributed leadership institutionalizes collaboration through role-based specialization: teaching–research group leaders and disciplinary pioneers transmute teacher interactions into organizational resource reservoirs [40]. These reservoirs exhibit dual characteristics—multilateral sharing exemplified by cross-grade lesson plan co-creation and intergenerational transmission evidenced by mentorship-driven pedagogical lineage preservation [41,42].

Teacher self-efficacy (TSE), functioning as psychological capital, arises from educators’ agentic conversion of organizational resources into personal competencies. A paradigmatic instance occurs in school-based curriculum development projects where teachers initially access institutional resources such as instructional design blueprints through collaborative networks, then progressively internalize these through reflective practice—transitioning from externally supported tools to internalized professional mastery [43,44]. This resource internalization process underscores the DL-COR nexus: distributed leadership’s institutional matrices not only consolidate critical resources like pedagogical expertise but also enable their dynamic reallocation through informal alliances, thereby mitigating uncertainties inherent in educational transformations.

### 2.2. The Relationship Between Distributed Leadership and Teacher Job Satisfaction

Distributed leadership embodies a horizontal, decentralized leadership paradigm marked by dynamic interactions between leaders, followers, and contextual factors, significantly impacting educational practices [22]. In China’s educational reforms, which emphasize school autonomy under centralized governance frameworks, distributed leadership serves as a strategic approach to reconciling top-down policy implementation with grassroots adaptability. While definitions of distributed leadership vary among scholars, consensus exists regarding its key elements: interactive leader–member relationships, role fluidity, shared responsibility and empowerment, and member engagement [45]. In practice, distributed leadership frequently takes the form of principals devolving authority to teachers, thus promoting extensive involvement in school-wide decision-making [10]. This paradigm shift aligns with China’s post-2019 educational reforms, particularly policy mandates advocating school autonomy and collective governance frameworks [46].

Empirical studies in Chinese contexts reveal that distributed leadership enhances teacher job satisfaction by addressing two systemic tensions: first, the reconciliation of standardized curriculum mandates with localized pedagogical innovations; second, the cultivation of teacher agency within hierarchical administrative structures [47,48]. Through empowering individuals, distributed leadership promotes organizational growth and generates significant benefits [49]. For instance, many schools institutionalize teacher leadership roles through mechanisms such as curriculum development committees and cross-departmental task forces [50]. As authority permeates the organization, teachers gain greater professional autonomy [51]. This process enables them to collaborate on the establishment of school development objectives [52,53,54,55]. Throughout this process, teachers gain enhanced recognition and appreciation, strengthening their sense of belonging and organizational identity [56].

In China’s collectivist culture, the emphasis on collective efficacy may exert a more pronounced effect on teacher job satisfaction compared to individualistic societies [30]. Moreover, principals’ intentional power-sharing in high power-distance contexts creates amplified psychological empowerment effects, as evidenced by teachers’ perceived elevation in professional status [57]. Such positive experiences boost teacher job satisfaction and motivate greater investment in teaching, which, in turn, enhances pedagogical autonomy and instructional quality [58,59,60,61,62]. Given these considerations, the present study posits Hypothesis 1:

**H1.**  *Distributed leadership shows a significant positive relationship with teacher job satisfaction.*

### 2.3. The Mediating Role of Teacher Collaboration

Teacher collaboration involves the exchange of ideas, professional dialogue on instructional practices, resource sharing, and collaborative endeavors among teachers [63]. This type of professional interaction, fostered within schools and among colleagues, represents a fundamental aspect of teacher collaboration [64]. Embedded in China’s education system, teacher collaboration is institutionalized through policy mechanisms such as teaching–research groups and mentorship systems, reflecting Confucian collectivist values that prioritize communal learning over individual competition.

Leadership structures critically shape teacher collaboration dynamics [21,65]. Under China’s decentralized governance framework, distributed leadership balances teacher empowerment with centralized accountability systems, including standardized school inspections and national curriculum requirements. By structurally integrating teachers into decision-making, distributed leadership enhances leadership capacity and fosters cooperative reform engagement [66]. This hybrid model fosters mutual trust and shared responsibility, enabling teachers to actively contribute to school reforms [20,32].

In education, teacher collaboration is recognized as a vital pathway for enhancing teaching quality and fostering professional development [64]. Research indicates that teachers engaging in collaborative activities typically demonstrate higher levels of confidence and job satisfaction compared to those working in isolation [67]. Institutional supports such as teaching–research groups align classroom practices with national reform goals, reducing role ambiguity and enhancing teacher job satisfaction. Moreover, in schools characterized by robust collaborative cultures, experience sharing and resource exchange improve work efficiency, reduce workloads, and promote pedagogical innovation [68,69,70,71]. These positive outcomes further elevate teachers’ satisfaction with their profession and work environment [72].

Crucially, the mediation between collaboration and job satisfaction is amplified by organizational support mechanisms and work environment alignment. Therefore, it can be inferred that the implementation of distributed leadership within schools can stimulate teacher collaboration, which, in turn, positively impacts teacher job satisfaction. Based on this analysis, the present study proposes Hypothesis 2:

**H2.**  *Teacher collaboration is a potential mediator in the association between distributed leadership and teacher job satisfaction.*

### 2.4. The Mediating Role of Teacher Self-Efficacy

Teacher self-efficacy denotes educators’ beliefs in their professional abilities and the effectiveness of their teaching methods, comprising three dimensions: instructional efficacy, classroom management efficacy, and student engagement efficacy [73]. In the Chinese educational context, teacher self-efficacy is further shaped by policy-driven reforms such as the “Double Reduction” initiative and culturally embedded professional practices, including structured collaborative lesson preparation and formal mentorship systems [74]. Teacher self-efficacy is markedly shaped by school leadership behaviors [75], particularly under distributed leadership models that align with China’s collectivist values.

According to the DL theory, when principals devolve decision-making authority and leadership duties to teachers, such as designing after-school services under policy mandates, it signals trust and acknowledges their professional competencies [76,77]. In China’s educational ecosystem, this empowerment enhances teachers’ psychological capital through institutional support from professional learning communities. In this setting, teachers benefit from organizational support and build psychological capital [78], leading to enhanced self-efficacy in organizational decision-making, interpersonal interactions, and teaching practices [79,80,81].

Empirical research consistently demonstrates a strong positive correlation between teacher self-efficacy and teacher job satisfaction [82], along with a notable negative correlation with occupational burnout [83]. Moreover, teacher self-efficacy is regarded as a critical determinant of professional well-being [84]. Teachers with elevated self-efficacy generally exhibit increased enthusiasm and job satisfaction [85,86].

Therefore, it can be inferred that distributed leadership enhances teacher self-efficacy, which, in turn, positively influences teacher job satisfaction. Based on this analysis, the present study proposes Hypothesis 3:

**H3.**  *Teacher self-efficacy is a potential mediator in the association between distributed leadership and teacher job satisfaction.*

### 2.5. The Chained Mediating Role of Teacher Collaboration and Teacher Self-Efficacy

The Conservation of Resources (COR) Theory posits that collaboration acts as a conditional resource embedded within institutionalized social interactions, such as school-based teaching–research groups with structured cooperation mechanisms, while teacher self-efficacy functions as a trait resource reflecting individual competencies [87,88]. COR theory emphasizes resource mobility, suggesting that external conditional resources (e.g., teacher collaboration) can integrate with individual trait resources (e.g., teacher self-efficacy) through resource compensation mechanisms and gain spirals [89,90]. This theoretical framework offers a foundation for comprehending how teacher collaboration can positively impact teacher self-efficacy.

The interaction between distributed leadership and COR theory manifests as a “structural empowerment-resource circulation” coupling path. Distributed leadership’s institutionalized structures provide organizational frameworks for COR-driven resource conservation, ensuring sustained accumulation of critical resources. Concurrently, informal collaborations redistribute resources to address environmental uncertainties. This constructive interaction is particularly evident in teacher professional development: distributed leadership enhances teacher self-efficacy by establishing stable resource allocation frameworks, while COR mechanisms further translate efficacy into job satisfaction, forming a “resource accumulation–psychological capital enhancement–satisfaction reinforcement” chain.

Research indicates that teacher collaboration significantly enhances teacher self-efficacy [24,91], with high-quality collaborative practices supporting teachers in acquiring solutions to instructional challenges and alternative experiences, thus promoting professional development and enhancing self-efficacy [92,93]. Based on this analysis, the present study proposes Hypothesis 4:

**H4.**  *Teacher collaboration and teacher self-efficacy form a sequential mediating path in the association between distributed leadership and teacher job satisfaction.*

**Figure 1:**
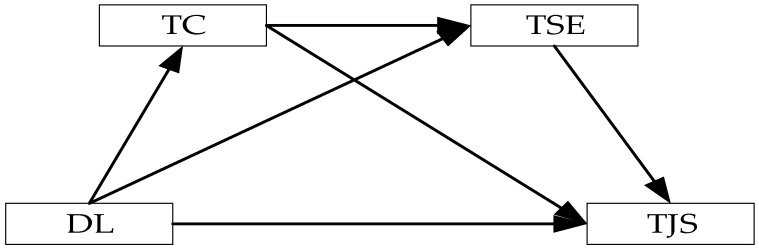
Theoretical Model of Distributed Leadership Impact on Teacher Job Satisfaction. DL: distributed leadership, TC: teacher collaboration, TSE: teacher self-efficacy, TJS: teachers job satisfaction.

In line with the theoretical explanations and hypotheses outlined above, this study develops a chained mediation model to elucidate how distributed leadership influences teacher job satisfaction via teacher collaboration and teacher self-efficacy. Specifically, this model posits that distributed leadership fosters teacher collaboration, which subsequently enhances teacher self-efficacy, resulting in higher teacher job satisfaction. The detailed pathways of this model are depicted in Figure 1.

## 3. Research Methodology

### 3.1. Data Source

The data for this study were obtained from TALIS 2018, administered by the OECD in that year. This extensive survey covered 31 OECD member countries and regions, as well as 17 non-OECD countries and regions, including China. Each participating country or region utilized a Probability Proportional to Size (PPS) sampling method, as specified by TALIS 2018 guidelines, to select sample schools [94].

In the Chinese context, the survey focused on Shanghai, distributing questionnaires to 4000 teachers across 200 sampled schools. Ultimately, 3976 valid responses were collected, representing 198 schools [95]. After excluding incomplete or missing data, the final analytical sample consisted of 3792 cases.

The sample characteristics show a gender distribution of 2791 female teachers (73.60%) and 1001 male teachers (26.40%), indicating a higher proportion of female teachers relative to the overall TALIS 2018 average of 68.30%.

Regarding educational background, most teachers possessed a bachelor’s degree (ISCED Level 6), numbering 3253 (85.78%), significantly higher than the TALIS 2018 average of 49.00%. Additionally, 507 teachers (13.37%) held a master’s degree or higher (Levels 7–8), slightly below the TALIS 2018 average of 44.00%, whereas only 32 teachers (0.84%) had an associate degree (Level 5).

In terms of teaching experience, the sample included 372 teachers (9.36%) with fewer than five years, 1539 teachers (38.71%) with 6–15 years, and 1881 teachers (47.31%) with more than 15 years. The mean teaching experience within the sample was 16.7 years, aligning with the overall TALIS 2018 average.

### 3.2. Research Variables

#### 3.2.1. Independent Variable

Distributed leadership (DL) functions as the independent variable in this study. The TALIS 2018 survey incorporates a distributed leadership scale consisting of three items (see Appendix A Table A1), including “This school has a culture of shared responsibility for school issues”. This scale utilizes a four-point Likert scale, where higher scores denote greater perceived levels of distributed leadership among teachers. Cronbach’s coefficient for this scale was 0.90, signifying excellent reliability.

Although distributed leadership is theoretically a team-level construct emerging from collective interactions, the TALIS Starting Strong 2018 technical report explicitly advises against using multilevel modeling due to the small number of staff within each center. To align with this data constraint, distributed leadership is operationalized in this study as individual teachers’ perceptions of leadership distribution within their school, measured through their Likert-scale responses to the distributed leadership items. This approach follows TALIS 2018’s analytical recommendations for contexts where cluster sizes are insufficient for reliable aggregation or multilevel analysis.

#### 3.2.2. Dependent Variable

Teacher job satisfaction serves as the dependent variable in this study. The TALIS 2018 survey assesses TJS using an eight-item scale segmented into two dimensions (see Appendix A Table A1). The Professional Satisfaction (PS) dimension comprises four items, including “Teaching offers more advantages than disadvantages”, whereas the Work Environment Satisfaction (WES) dimension encompasses four items, such as “If I had the choice again, I would not change schools”. Similarly, this scale employs a four-point Likert scale, where higher scores reflect greater teacher job satisfaction. The overall Cronbach’s α coefficient for the teacher job satisfaction scale was 0.84, with subscale coefficients of 0.75 for professional satisfaction and 0.74 for WES, indicating satisfactory reliability.

#### 3.2.3. Mediating Variables

Teacher collaboration functions as a mediating variable in this study. The TALIS 2018 survey assesses teacher collaboration using an eight-item scale segmented into two dimensions (see Appendix A Table A1). The Professional Collaboration (PC) dimension encompasses four items, including “Engagement in collaborative professional learning activities (e.g., teaching research activities)”, whereas the Coordination for Teaching dimension also consists of four items, such as “Teach jointly as a team in the same class”. This scale utilizes a six-point Likert scale, where higher scores denote greater levels of teacher collaboration. The Cronbach’s α coefficient for the overall teacher collaboration scale was 0.85, with subscale coefficients of 0.70 for Professional collaboration and 0.82 for coordination for teaching, signifying satisfactory reliability.

TSE also functions as a mediating variable in this study. The TALIS 2018 survey evaluates TSE using a twelve-item scale segmented into three dimensions (see Appendix A Table A1). The Classroom Teaching Efficacy (CTE) dimension comprises four items, including “Offering alternative explanations when students are perplexed”, the Classroom Management Efficacy (CME) dimension encompasses four items, such as “Ensuring students adhere to classroom rules”, and the Student Engagement Efficacy (SEE) dimension consists of four items, like “Persuading students of their ability to succeed in their studies”. This scale employs a four-point Likert scale, where higher scores indicate greater levels of TSE. The overall Cronbach’s α coefficient for the TSE scale was 0.96, with subscale coefficients of 0.90 for CTE, 0.92 for CME, and 0.90 for student engagement efficacy, signifying excellent reliability.

#### 3.2.4. Control Variables

Empirical research suggests that gender, educational attainment (EA), and teaching experience (TE) can impact teacher job satisfaction [96,97]. To ensure the accuracy and robustness of the study’s findings, these factors are incorporated as control variables in this analysis.

### 3.3. Data Analysis Methods

This study utilized SPSS 27.0 and AMOS 26.0 for data analysis. SPSS 27.0 was employed for reliability testing, assessing common method bias, evaluating discriminant validity, conducting descriptive statistical analysis, and performing correlation analysis to ensure the robustness and validity of the data. AMOS 26.0 was used to construct structural equation models (SEM) to examine the relationship between distributed leadership and teacher job satisfaction and explore the potential associations mediated by teacher collaboration and teacher self-efficacy. To further validate the significance of the mediation effects, this study applied the Bootstrap Method for testing.

## 4. Research Results

### 4.1. Common Method Bias and Discriminant Validity Testing

To mitigate potential common method bias, this study conducted Harman’s single-factor test to assess the core variables. This analysis revealed six common factors with eigenvalues exceeding one, with the initial factor accounting for 30.71% of the total variance, which falls below the critical threshold of 40%, indicating a lack of significant common method bias. However, acknowledging the limited sensitivity of the Harman test [80], this study additionally performed a single-factor confirmatory factor analysis (CFA). The results demonstrated a poor model fit (χ^2^ = 33,701.853, df = 434, *p* > 0.05, χ^2^/df = 77.654, RMSEA = 0.142, GFI = 0.504, CFI = 0.541, RMR = 0.181, NFI = 0.538, NNFI = 0.509), thereby reinforcing the conclusion that common method bias is not significant.

Furthermore, the discriminant validity among the variables was assessed via CFA. As illustrated in Table 1, the fit indices of the four-factor baseline model were markedly superior to those of alternative models, signifying that the four-factor model more accurately reflects the underlying factor structure. This outcome confirms robust discriminant validity among the four core variables.

### 4.2. Descriptive Statistics and Correlation Analysis of Variables

The means, standard deviations, and results of the correlation analysis for distributed leadership, teacher collaboration, teacher self-efficacy, and teacher job satisfaction are summarized in Table 2. The correlation analysis indicates that all variables display significant positive correlations with one another. Specifically, distributed leadership, teacher collaboration, and teacher self-efficacy exhibit positive correlations with teacher job satisfaction. These findings are consistent with theoretical expectations and suggest the potential for conducting further in-depth analyses.

### 4.3. Chain Mediation Model Testing

In line with the proposed hypotheses, this study developed an SEM to examine the impact of distributed leadership on teacher job satisfaction. Within this model, distributed leadership functions as the independent variable, and teacher job satisfaction as the dependent variable, while teacher collaboration and teacher self-efficacy serve as mediating variables. Additionally, gender, educational attainment, and teaching experience were incorporated as control variables for all endogenous variables (TC, TSE, TJS) to account for potential confounding effects.

The analysis revealed that neither gender nor educational attainment significantly predicted teacher collaboration (TC) (β = −0.012, *p* = 0.412; β = 0.021, *p* = 0.227), whereas teaching experience (TE) exhibited a significant negative association with TC (β = −0.038, *p* = 0.001). Similarly, gender and educational attainment did not show significant effects on teacher self-efficacy (TSE) (β = −0.008, *p* = 0.561; β = 0.015, *p* = 0.389), while TE remained negatively associated with TSE (β = −0.029, *p* = 0.003). This suggests that more experienced teachers may perceive reduced collaboration and self-efficacy within distributed leadership frameworks.

The SEM results demonstrate that the factor loadings for each measured construction ranged from 0.654 to 0.924, signifying strong explanatory power. Moreover, the model fit indices were exemplary: RMSEA = 0.031, GFI = 0.994, CFI = 0.995, and NFI = 0.994, all falling within the recommended thresholds. These findings confirm the well-fitting nature of the constructed SEM.

**Figure 2:**
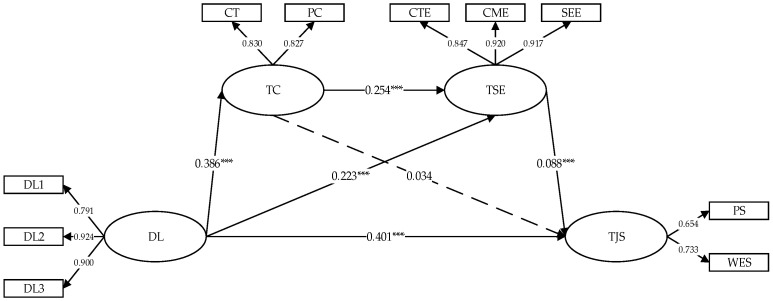
Path Diagram of distributed leadership’s Impact on teacher job satisfaction. *** *p* < 0.001. DL: distributed leadership, TC: teacher collaboration, TSE: teacher self-efficacy, TJS: teachers job satisfaction, CT: coordination for teaching, PC: professional collaboration, CTE: classroom teaching efficacy, CME: classroom management efficacy, SEE: student engagement efficacy, PS: professional satisfaction, WES: work environment satisfaction.

The standardized path coefficients depicted in Figure 2 indicate that distributed leadership exerts a significant positive direct influence on teacher job satisfaction (β = 0.401, *p* < 0.01), thereby corroborating Hypothesis H1. Furthermore, distributed leadership positively impacts both teacher collaboration (β = 0.386, *p* < 0.01) and TSE (β = 0.223, *p* < 0.01). Teacher collaboration positively affects TSE (β = 0.254, *p* < 0.01); however, its influence on teacher job satisfaction is not statistically significant (β = 0.034, *p* > 0.1). Conversely, TSE significantly and positively influences teacher job satisfaction (β = 0.088, *p* < 0.01).

To further validate the mediating effects, this study utilized a bias-corrected percentile Bootstrap Method with 1000 resamples to evaluate the mediating roles of teacher collaboration and teacher self-efficacy in the relationship between distributed leadership and teacher job satisfaction, as outlined in Table 3. The findings indicate that the direct effect of distributed leadership on teacher job satisfaction is robust, with an effect size of 0.401 and a 95% confidence interval [0.12, 0.15], which excludes zero. This confirms the statistical significance of the direct path from distributed leadership to teacher job satisfaction, thereby supporting Hypothesis H1.

In contrast, the indirect effect of teacher collaboration on the relationship between distributed leadership and teacher job satisfaction was found to be minimal, with an effect size of 0.013 and a 95% confidence interval [−0.01, 0.04], which includes zero. This suggests that teacher collaboration does not act as a statistically significant mediator in the proposed relationship, failing to support Hypothesis H2.

Alternatively, teacher self-efficacy demonstrates a significant mediating effect, with an effect size of 0.019 and a 95% confidence interval [0.03, 0.06], which excludes zero. This indicates that TSE plays a crucial mediating role, contributing 5.56% to the total effect, thereby supporting Hypothesis H3.

Moreover, the combined indirect effect of teacher collaboration and teacher self-efficacy on the relationship between distributed leadership and teacher job satisfaction is also significant, with an effect size of 0.009 and a 95% confidence interval [0.01, 0.02], which excludes zero. This indicates that teacher collaboration and teacher self-efficacy together exhibit a sequential mediation effect, contributing 1.85% to the total effect, thus validating Hypothesis H4.

## 5. Discussion

This study examined the association between distributed leadership and teacher job satisfaction within the context of the Chinese educational system while exploring the potential mediating roles of teacher collaboration and TSE. Empirical findings indicate a robust and significant positive relationship between distributed leadership and teacher job satisfaction among educators in China, which is consistent with prior findings that distributed leadership fosters teacher job satisfaction in different countries and regions [17,24]. Additionally, a key finding is that TSE serves as a distinct mediator, emerging as a crucial element in the examined relationship, whereas teacher collaboration does not independently mediate the relationship. Furthermore, evidence supports a chain mediation role, wherein the combined association of teacher collaboration and TSE strengthens the link between distributed leadership practices and enhanced teacher job satisfaction.

### 5.1. Association Between Distributed Leadership and Teacher Job Satisfaction

The empirical findings demonstrate that distributed leadership is strongly associated with higher teacher job satisfaction among Chinese teachers. This is consistent with the research by Hulpia [32] and Torres [17], who reported analogous associations in Western contexts, thereby reinforcing the cross-cultural relevance of distributed leadership. By promoting the distribution of power, shared accountability, and decentralized leadership, this approach empowers teachers with greater authority and participation in decision-making, which enhances their sense of ownership and affiliation and fosters positive professional dispositions.

Nevertheless, it is important to note that our results differ from the results of a Japanese study, where the direct association between distributed leadership and teacher job satisfaction was found to be insignificant [98]. Some researchers posit that individuals from East Asian cultures, which are typified by collectivist values, might have less pronounced demands for personal autonomy [99,100]. However, our investigation indicates that the importance of individual autonomy persists even in collectivist cultures. This finding highlights the importance of avoiding overgeneralizations about cultural traits and underscores the need to explore the diversity and intricacies within various cultures when examining leadership models.

As society evolves and individual consciousness increases, Chinese teachers’ aspirations for professional growth have escalated, resulting in a heightened demand for greater autonomy and opportunities for self-expression. Furthermore, the Chinese government is actively advancing the modernization of educational administration and enhancing school management standards [84]. In this context, distributed leadership is garnering increasing recognition and is progressively supplanting traditional “heroic” leadership paradigms [85]. The empirical results affirm that this management approach has achieved remarkable success in the Chinese educational sector.

The findings are embedded within the cultural context of collectivism and high-power distance inherent in Chinese educational institutions, which may constrain the generalizability of the sequential mediation mechanism to other cultural systems. In societies marked by low power distance and individualistic values—for instance, Canada and America—the mediating role of teacher collaboration may diminish due to educators’ heightened autonomy in defining collaborative practices [29]. By contrast, the teacher self-efficacy pathway could gain prominence in such contexts, as self-efficacy development in individualistic cultures often prioritizes personal agency over institutional support [101]. These propositions resonate with cross-cultural research, underscoring that the efficacy of leadership practices on teacher outcomes depends on their alignment with dominant cultural norms [102].

### 5.2. Independent Mediating Role of Teacher Self-Efficacy and Teacher Collaboration

This study demonstrates that teacher self-efficacy acts as a significant independent mediator between distributed leadership and teacher job satisfaction, a result that aligns with Sun [103]. As an integral component of positive psychological capital [87], teacher self-efficacy is a crucial factor influencing teacher job satisfaction [82]. Distributed leadership, through its empowering behaviors, instills trust and provides support, thereby strengthening teachers’ confidence in their roles and their conviction in managing teaching challenges effectively [81].

Teachers with high self-efficacy demonstrate greater work motivation and involvement, actively engaging in teaching activities and seeking out opportunities for professional development [104]. They are more inclined to refine their teaching philosophies and methodologies, deriving increased personal and professional satisfaction from their work, which ultimately enhances their job satisfaction [85]. Furthermore, high self-efficacy equips teachers with more effective strategies to mitigate occupational burnout, allowing them to implement robust coping mechanisms under stress, thereby safeguarding job satisfaction from burnout-related declines [105,106]. Consequently, distributed leadership indirectly improves teacher job satisfaction by bolstering self-efficacy, highlighting the central role of self-efficacy in professional development and overall job performance.

Teacher collaboration did not independently mediate the relationship between distributed leadership and teacher job satisfaction, a result that differs from the findings of Torres et al. [21,71]. This discrepancy may be attributed to the differing cultural and systemic contexts in which these studies were conducted. In Asian countries with Confucian heritage, collaborative learning within a collectivist framework is frequently met with skepticism and resistance [107]. Additionally, the variations in cultural and educational systems lead to differences in cooperative behaviors and outcomes among teachers in Eastern and Western settings [67].

In China, the cultural context for teacher collaboration is characterized by unique nuances. First, Confucianism emphasizes hierarchical structures and respect for authority, which shape the dynamics of teacher collaboration through high power distance [31]. Although distributed leadership seeks to enhance collaboration by decentralizing authority, teachers might still prioritize compliance with directives from superiors over proactive engagement in collaborative efforts. Second, evaluation mechanisms in the Chinese education system tend to emphasize individual performance metrics, relegating collaboration to a secondary role in assessments and evaluations [108]. This institutional design can potentially undermine teachers’ enthusiasm for collaborative activities, especially when combined with heavy workloads that leave little time or energy for meaningful cooperation [109]. Moreover, the effectiveness of teacher collaboration depends on several factors, such as mutual trust, communication skills, and clearly defined collaborative goals [110,111].

Nevertheless, this should not be construed as suggesting that teacher collaboration holds no value within the context of distributed leadership. Studies have shown that improved teacher collaboration has a positive impact on teacher job satisfaction [112]. Distributed leadership fosters positive interpersonal relationships and a sense of belonging, which can lead to increased teachers’ satisfaction with their profession and work environment [113]. Therefore, educational administrators ought to prioritize fostering a collaborative ethos by creating environments and conditions conducive to effective teacher collaboration. By doing so, teachers can benefit from increased support and professional development opportunities through collaboration, ultimately resulting in higher job satisfaction.

### 5.3. Chain Mediation Role of Teacher Collaboration and Teacher Self-Efficacy

The findings indicate that teacher collaboration and teacher self-efficacy form a sequential indirect pathway in the relationship between distributed leadership and teacher job satisfaction. Specifically, the statistical model suggests that distributed leadership is linked to higher teacher collaboration, which, in turn, is associated with higher teacher self-efficacy, and both are collectively related to greater teacher job satisfaction. This finding provides a deeper understanding of the mechanisms through which distributed leadership operates within the collectivist context of China.

Distributed leadership promotes a supportive and democratic work environment by affording teachers greater autonomy and decision-making power. According to the reciprocity principle [114], teachers are likely to reciprocate principals’ support with collaborative endeavors. From the perspective of resource dependency theory [87], collaboration serves as a contingent resource, and self-efficacy develops as a personal asset grounded in this foundation [88]. Through collaboration, teachers acquire valuable teaching experiences and feedback, thereby strengthening their competencies and reinforcing their self-efficacy, which contributes to higher job satisfaction [21,69,92].

The chain mediation mechanism identified in this research offers valuable insights for educational administrators. First, it underscores the necessity to prioritize the organization and facilitation of teacher collaboration. Administrators should create opportunities and platforms that foster collaboration, along with establishing robust support mechanisms. Second, attention must be directed toward cultivating teacher self-efficacy. Through targeted training programs and incentive structures, administrators can strengthen teachers’ professional competencies and boost their confidence. Moreover, enhancing teacher job satisfaction is a systemic endeavor that necessitates a holistic consideration of the interplay between distributed leadership, teacher collaboration, and TSE. Only through an integrated approach that harmonizes these elements can the full potential of distributed leadership be fully realized.

### 5.4. Limitations

The present study has two principal limitations. First, the data are drawn solely from TALIS 2018 conducted in Shanghai, which may not fully encapsulate the variability in how distributed leadership affects teacher job satisfaction across diverse regions within mainland China. This geographical focus limits the generalizability of the results. Future research might utilize stratified cluster sampling methods to obtain a wider and more representative sample, thus offering richer insights into the efficacy of distributed leadership practices across varied regional contexts.

Second, the interaction between principals and teachers is intrinsically dynamic, with teachers’ attitudes and behaviors likely to evolve in response to shifts in leadership styles and work environments. However, the cross-sectional nature of the dataset used does not adequately capture these evolving dynamics. Consequently, future studies could incorporate longitudinal tracking or experimental designs to examine the temporal impact of distributed leadership on teacher job satisfaction more thoroughly.

Third, the cross-sectional design inherently limits the establishment of temporal precedence in mediation mechanisms. Although structural equation modeling and bootstrap methods revealed statistically significant indirect associations between distributed leadership and teacher job satisfaction through teacher collaboration and teacher self-efficacy, these findings should be interpreted as exploration evidence of potential pathways, not as confirmed causal mediation. Future studies could adopt longitudinal designs to evaluate whether distributed leadership causally influences teacher job satisfaction through the proposed sequential mediators over time.

## 6. Conclusions

This study investigated the influence of distributed leadership on teacher job satisfaction within the Chinese educational system and examined its underlying mechanisms. The findings demonstrate that distributed leadership has a significant positive effect on teacher job satisfaction. Teacher self-efficacy acts as an independent mediator between distributed leadership and teacher job satisfaction, whereas teacher collaboration combined with teacher self-efficacy exerts a chain mediation influence. These insights enhance our understanding of the mechanisms through which distributed leadership operates and underscore the significance of fostering self-efficacy and encouraging collaborative practices among teachers. This study offers practical implications for educational administrators in China who wish to implement distributed leadership and contributes to the body of the literature on this leadership approach.

## Figures and Tables

**Table 1:** Comparison of Measurement Models.

Model	χ^2^	*df*	*p*	χ^2^/df	RMSEA	GFI	CFI	RMR	NFI	NNFI
Benchmark Model	9357.268	428	0	21.863	0.074	0.843	0.877	0.037	0.872	0.866
Model 1	16,660.335	431	0	38.655	0.099	0.747	0.776	0.053	0.772	0.759
Model 2	17,419.515	431	0	40.417	0.102	0.747	0.766	0.059	0.761	0.747
Model 3	18,168.513	431	0	42.154	0.104	0.681	0.756	0.178	0.751	0.736
Model 4	25,977.731	433	0	59.995	0.125	0.608	0.648	0.179	0.644	0.622

Benchmark Model: Distributed Leadership (DL), Teacher Collaboration (TC), Teacher Self-Efficacy (TSE), and Teachers’ Job Satisfaction (TJS); Model 1: DL + TC, TSE, TJS; Model 2: DL + TSE, TC, TJS; Model 3: TC + TSE, DL, TJS; Model 4: DL + TC + TSE, TJS.

**Table 2:** Descriptive Statistics and Correlation Analysis.

Variables	Mean	SD	1	2	3	4
1. DL	3.052	0.602	1			
2. TC	3.812	0.969	0.334 ***	1		
3. TSE	3.311	0.542	0.296 ***	0.297 ***	1	
4. TJS	2.900	0.465	0.492 ***	0.254 ***	0.266 ***	1

*** *p* < 0.001.

**Table 3:** Results of Chain Mediation Testing for teacher collaboration and TSE.

Path Description	Effect Size (β)	95% CI		Proportion of Total Effect
		Lower Bound	Upper Bound	
Total Effect	0.442	0.16	0.21	100%
Direct Effect	0.401	0.12	0.15	90.12%
Indirect Effect	0.041	0.05	0.10	9.88%
DL→TC→TJS	0.013	−0.01	0.04	2.47%
DL→TSE→TJS	0.019	0.03	0.06	5.56%
DL→TC→TE→TS	0.009	0.01	0.02	1.85%

## Data Availability

The data are from the TALIS 2018 Database. https://webfs.oecd.org/talis/TALIS-Starting-Strong-By-country-SPSS.zip (accessed on 10 December 2024).

## Abbreviations

The following abbreviations are used in this manuscript:

CFA	Confirmatory factor analysis
CME	Classroom management efficacy
COR	Conservation of resources
CT	Coordination for teaching
CTE	Classroom teaching efficacy
DL	Distributed leadership
EA	Educational attainment
PC	Professional collaboration
PPS	Probability proportional to size
PS	Professional satisfaction
SEE	Student engagement efficacy
SEM	Structural equation models
TALIS	Teaching and Learning International Survey
TC	Teacher collaboration
TE	Teaching experience
TJS	Teacher job satisfaction
TSE	Teacher self-efficacy
WES	Work environment satisfaction

## Appendix A

**Table A1:** Item Wording for Scales.

**Scale**	**Subscale**	**Item**	**Response Option**
DL		This school provides staff with opportunities to actively participate in school decisions. This school has a culture of shared responsibility for school issues. There is a collaborative school culture that is characterized by mutual support.	(1) Strongly Disagree; (2) Disagree; (3) Agree; (4) Strongly Agree.
TJS	PS	The advantages of being a teacher clearly outweigh the disadvantages. If I could decide again, I would still choose to work as a teacher. I regret that I decided to become a teacher. I wonder whether it would have been better to choose another profession.	(1) Strongly Disagree; (2) Disagree; (3) Agree; (4) Strongly Agree.
	WES	I would like to change to another school if that were possible. I enjoy working at this school. I would recommend this school as a good place to work. All in all, I am satisfied with my job.	
TC	PC	Teach jointly as a team in the same class. Observe other teachers’ classes and provide feedback. Engage in joint activities across different classes and age groups (e.g., projects). Take part in collaborative professional learning.	(1) Never; (2) Once a year or less; (3) 2–4 times a year; (4) 5–10 times a year; (5) 1–3 times a month; (6) Once a week or more.
	CT	Teach jointly as a team in the same class. Exchange teaching materials with colleagues. Engage in discussions about the learning development of specific students. Work with other teachers in this school to ensure common standards in evaluations for assessing student progress.	
TSE	CTE	Craft good questions for students. Use a variety of assessment strategies. Provide an alternative explanation, for example, when students are confused. Vary instructional strategies in my classroom.	(1) Not at all; (2) To some extent; (3) Quite a bit; (4) A lot.
	CME	Control disruptive behavior in the classroom. Make my expectations about student behavior clear. Get students to follow classroom rules. Calm a student who is disruptive or noisy.	
	SEE	Get students to believe they can do well in school work. Help students value learning. Motivate students who show low interest in school work. Help students think critically.	
